# The Synthetic Antimicrobial Peptide 19-2.5 Interacts With Heparanase and Heparan Sulfate in Murine Sepsis *In Vivo* and in Human Sepsis *Ex Vivo*

**DOI:** 10.1186/2197-425X-3-S1-A516

**Published:** 2015-10-01

**Authors:** L Martin, S Doemming, A Humbs, L Heinbockel, K Brandenburg, G Marx, T Schürholz

**Affiliations:** Department of Intensive Care and Intermediate Care, University Hospital RWTH, Aachen, Germany; Division of Biophysics, Forschungszentrum, Borstel, Germany

## Introduction

Heparanase is an endo-β-glucuronidase that cleaves highly potent heparan sulfate (HS) from its proteoglycan, thereby triggering the inflammatory response in [1]. Thus, new anti-infective agents that interact with heparanase may be promising tools for sepsis therapy. As a novel anti-infective agent, peptide 19-2.5 (pep2.5) belongs to the class of synthetic anti-lipopolysaccharide peptides, however its activity is not restricted to Gram-negative bacterial infection [2, 3].

## Objectives

To evaluate the interaction of pep2.5 with heparanase in murine sepsis *in vivo* and in human sepsis *ex vivo*.

## Methods

First, we used a model of murine cecal ligature and puncture (CLP) sepsis to study the impact of pep2.5 on heparanase *in vivo* in 12 NMRI mice. Mice were treated with pep2.5 or NaCl 0.9%. Plasma was sampled 24h after CLP. Second, we investigated whether pep2.5 interacts with heparanase in human plasma samples *ex vivo*. We added pep2.5 (20µg/ml) to plasma of 18 septic shock patients according to the ACCP/SCCM definitions and to plasma of 10 healthy volunteers. Heparanase-levels, HS-levels and heparanase activity were measured using ELISA (AMS Biotechnology, Oxon, United Kingdom). All data are given as mean ± standard deviation. a t-test with Holm-Šídák correction was used and a p-value < 0.05 was considered significant.

## Results

**Figure 1:**
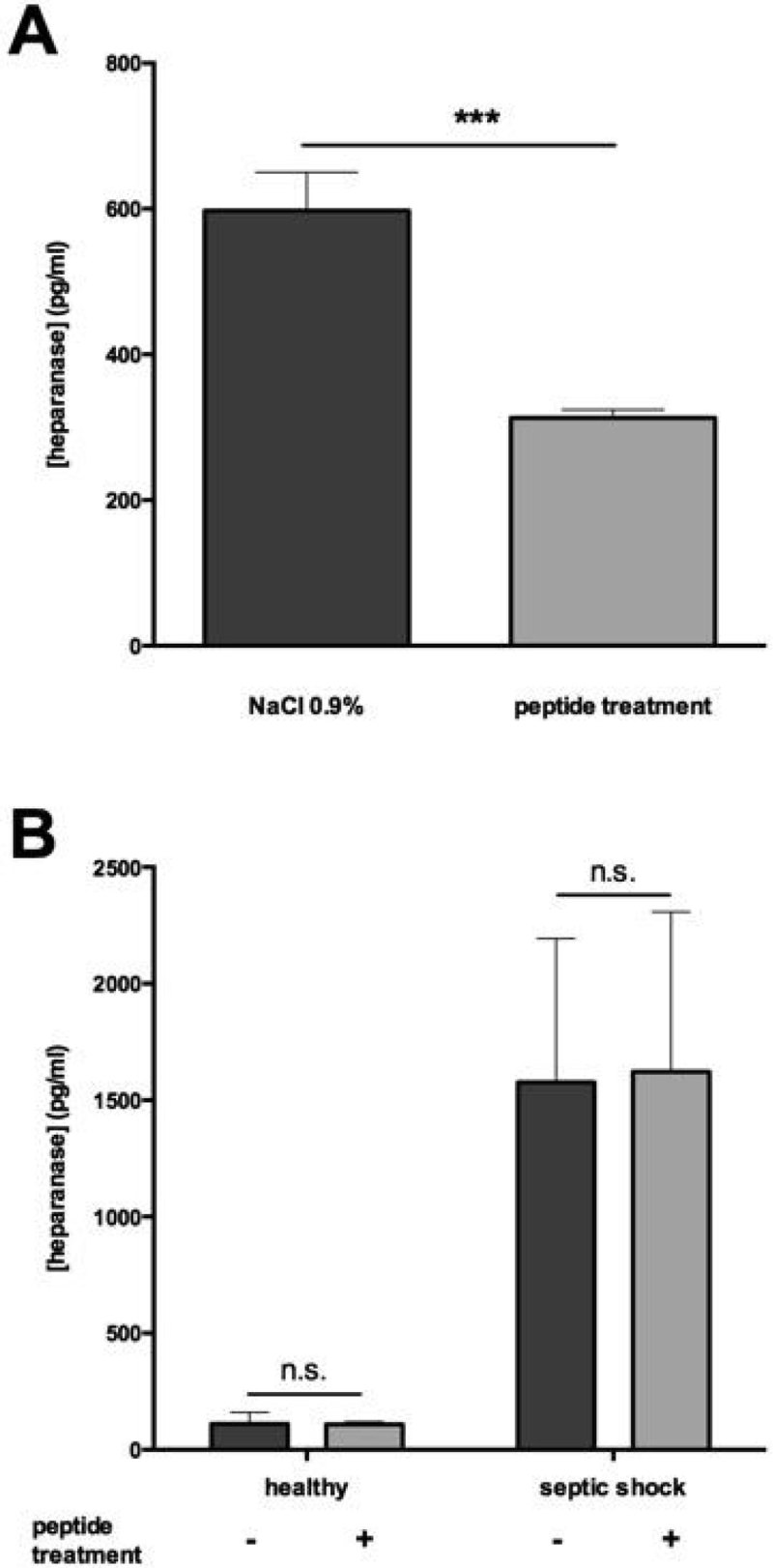
(**A**) Heparanase level in CLP-mice. (**B**) Heparanase level in human.*p < 0.05, **p < 0.005, ***p < 0.001.

Mice subjected to CLP without treatment displayed higher heparanase levels in plasma compared to mice treated with pep2.5 (p < 0.0001). Treatment with pep2.5 resulted in lower heparanase activity (p < 0.0001) and reduced HS-levels (p < 0.0001), compared to untreated animals (Figure 1).

Septic shock patients (78% male) were 70 ± 15 years old and healthy volunteers (50% male) were 67 ± 19 years old. Plasma heparanase levels, heparanase activity and HS-levels were significantly higher in individuals with septic shock than in healthy individuals (all p < 0.0001). the *ex vivo* addition of pep2.5 did not impact heparanase levels, however heparanase activity and HS-levels were decreased by adding pep2.5 to plasma of septic shock patients (all p < 0.05, Figure 1).

## Conclusions

The synthetic antimicrobial peptide 19-2.5 interacts with heparanase in human and murine sepsis and reduces levels of highly potent HS. Thus, peptide 19-2.5 may have the potential for further development as a new anti-infective drug in sepsis therapy.

## Grant Acknowledgment

This work was supported by an intramural grant to Dr. Lukas Martin (START 693900).

**Figure 2:**
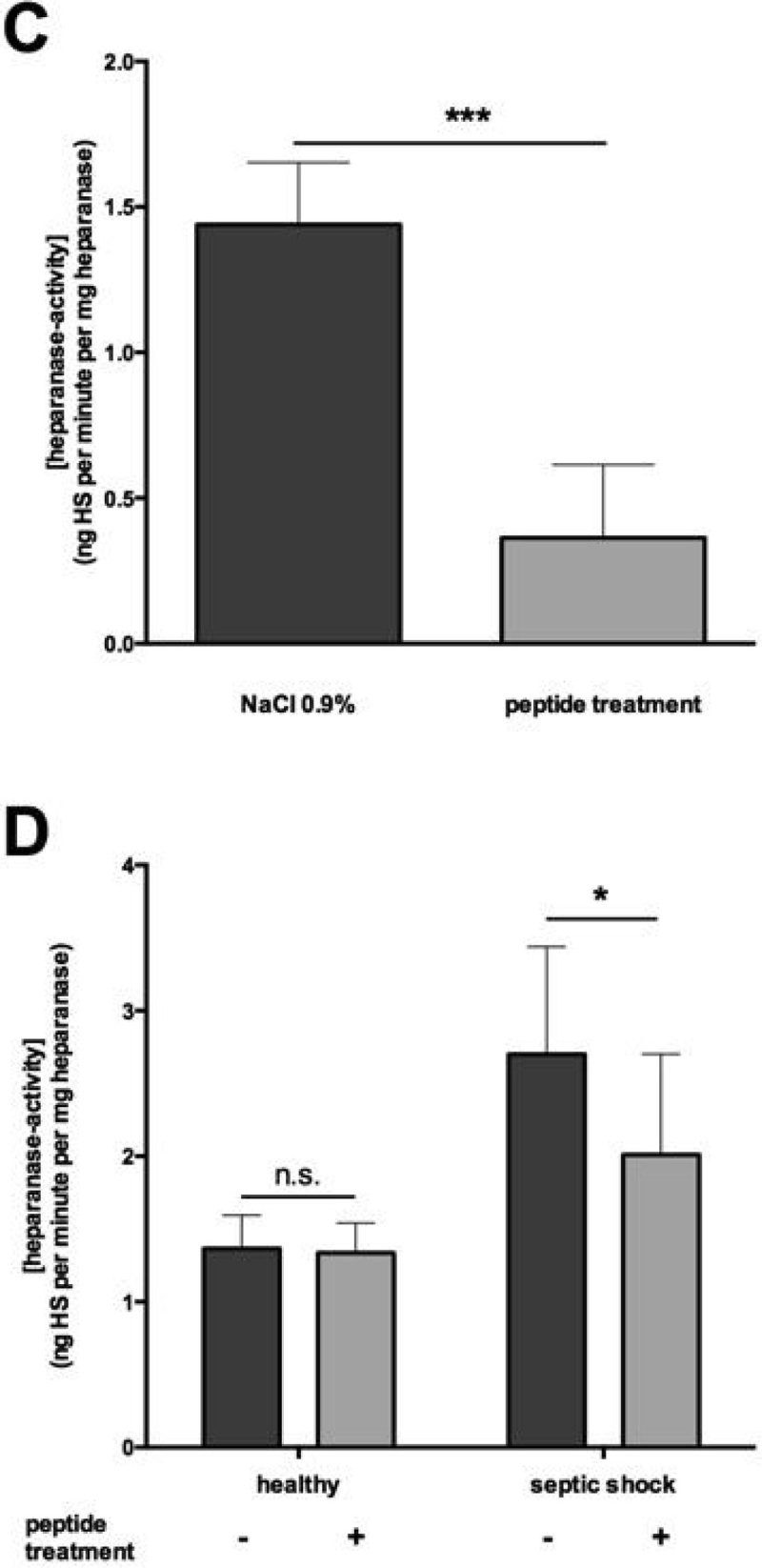
(**C**) Heparanase activity in CLP-mice. (**D**) Heparanase activity in human. *p < 0.05, **p < 0.005, ***p < 0.001.

**Figure 3:**
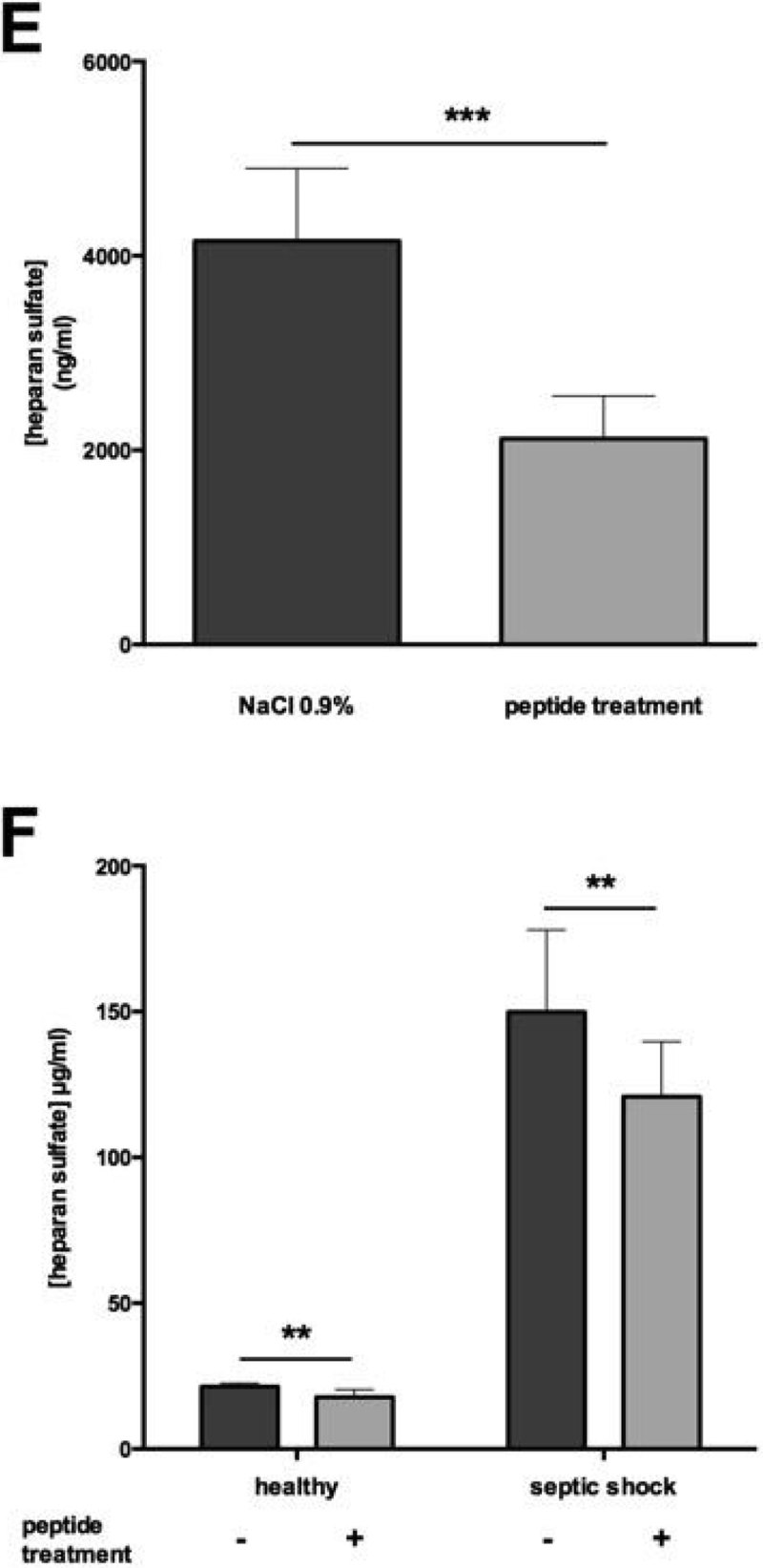
(**E**) Heparan sulfate level in CLP-mice. (**F**) Heparan sulfate level in human. *p < 0.05, **p < 0.005, ***p < 0.001.
